# Comparison of lateral approach versus anterolateral approach with Herbert screw fixation for isolated coronal shear fractures of humeral capitellum

**DOI:** 10.1186/s13018-019-1261-3

**Published:** 2019-07-22

**Authors:** Tengbo Yu, Hao Tao, Fenglei Xu, Yanling Hu, Chengdong Zhang, Guangjie Zhou

**Affiliations:** 1 Department of Orthopaedic Surgery, Affiliated Hospital of Qingdao University, 59 Haier Road, Qingdao, Shandong 266003 People’s Republic of China; 2 Department of Radiology, Affiliated Hospital of Qingdao University, Qingdao, Shandong 266000 People’s Republic of China

**Keywords:** Capitellum, Fracture, Surgical approach, Internal fixation, Herbert screw

## Abstract

**Background:**

For coronal shear fractures of humeral capitellum, the lateral approach is the most commonly used surgical approach. However, exposure range of the anterior aspect of the distal humerus is inadequate. The anterolateral approach has also been adopted to overcome this disadvantage. However, this approach seems anatomically complex due to the risk of iatrogenic injury to the radial nerve. So far, the optimal approach for the treatment of capitellar shear fractures remains inconclusive. The purpose of this study is to prospectively review and compare the early clinical and radiographic outcomes of treated with open reduction and Herbert screw internal fixation through the lateral approach or the anterolateral approach.

**Methods:**

Twenty-six patients with isolated capitellar shear fractures were enrolled from January 2013 to December 2017, and randomly assigned to lateral approach group or anterolateral approach group. All the fractures were treated with open reduction and Herbert screw internal fixation through lateral approach or anterolateral approach. Operation time, wound healing complication, elbow joint function, and radiographic evidence were evaluated and compared between two groups.

**Results:**

The operation via the anterolateral approach took significantly shorter time than via lateral approach (*p* < 0.05). There were no wound healing problems and infection for both groups. One patient from anterolateral approach group sustained incomplete posterior interosseous nerve palsy, which recovered completely in 4 weeks without residual compromise. All fractures healed well in their normal anatomic position as seen on radiographs. At the final follow-up, no significant difference was found between two groups with respect to the ROM in supination-pronation, ROM in pronation-supination, loss of flexion-extension motion, or loss of pronation-supination motion (*p* > 0.05). There is no significant difference with respect to MEPI score of elbow joint between two groups (*p* > 0.05).

**Conclusion:**

Based on our findings, both lateral approach and anterolateral approach with Herbert screw internal fixation are suitable for coronal shear fractures of capitellum with satisfactory early outcomes. Compared with the lateral approach, the anterolateral approach made the surgical procedure easier and time saving in current series. When the medial aspect of the trochlea is involved for capitellar coronal fractures, the anterolateral lateral approach should be preferred.

## Background

Fractures of the humeral capitellum are rare injuries [1]. Due to the intraarticular and complex nature of these injuries and their rarity, it has been technically challenging to treat these injuries [2–4]. Inadequately treated fractures may lead to a severe compromise in function owing to the restricted range of motion (ROM). Currently, open reduction and internal fixation with an aim to provide stable and congruent joint has been considered to be a standard treatment [5].

The choice of surgical approach is one of the main areas of interest in the surgical management of fractures. For coronal shear fractures of capitellum, namely capitellar fractures without involvement of posterior aspect, most reports have used the lateral approach of elbow joint [6–16]. Though favorable outcomes have been reported, exposure range of the anterior aspect of the distal humerus is inadequate through this approach. Benefiting from excellent visualization and allowing access to easier perpendicular fracture screw fixation, the anterolateral approach of elbow joint has also been adopted to treat this type of fracture in several reports [17–21]. However, this approach carries a risk of iatrogenic injury to the radial nerve.

So far, the optimal approach for the treatment of capitellar shear fractures remains inconclusive. In this study, we aimed to prospectively review and compare the early clinical and radiographic outcomes of this kind of fractures treated with open reduction and internal Herbert screw fixation through the lateral approach or the anterolateral approach. To the best of our knowledge, this is the first report of such a comparison.

## Materials and methods

### Patients

From January 2013 to December 2017, 32 consecutive patients with isolated capitellar shear fractures were collected from our hospital. Plain radiographs and computed tomography scans with a three-dimensional reconstruction were performed routinely to better define fracture lines and rule out associated injuries, such as the coronoid process fractures, a dislocation or injury to the radial head, epicondylar avulsion fractures, or elbow dislocations.

Fractures were classified according to the Dubberley classification system [2]. Type 1 is a fracture involving primarily the capitellum with or without the lateral trochlear ridge. Type 2 is a fracture involving the capitellum and the trochlea as one piece. Type 3 is a fracture involving both the capitellum and the trochlea as separate fragments. These fractures were further classified as type A and type B based on the absence or presence of posterior condylar comminution. Type B fractures were excluded because these fractures may be treated through a posterior approach due to the presence of a posterior condylar comminution. All fractures in the current series were coronal shear fractures without involvement of posterior aspect of capitellum. Patients were also excluded if they had one of the following: age ≤ 18 or ≥ 65 years old; decline to participate; old fractures over 14 days; brain trauma; diabetes, rheumatoid arthritis, or neurologic disorders. Thus, 29 patients were included in the study.

The study was a prospective, single-blind, randomized trial. This study was approved by the Institutional Review Board of the Affiliated Hospital of Qingdao University. Informed consent was obtained. The patients were randomly allocated by drawing lots to either lateral approach group (via lateral approach) or anterolateral approach group (via anterolateral approach). All operations were performed by two senior orthopedic surgeons (YL Hu and TB Yu) who were experienced in surgical procedure for elbow joint fractures and dislocations. Immediately after surgery, three patients were excluded. One patient underwent excision of the capitellar fragments because of insufficient subchondral bone and comminution of the articular segment. Two patients were found to be combined with collateral ligament injuries through intraoperative physical examination. After fracture fixation, ligament repair was performed. Finally, a total of 26 patients with isolated capitellar shear fractures were enrolled in the study.

### Operation procedure

All the patients in both groups were administered a brachial plexus anesthesia and placed in the supine position with a tourniquet on the upper arm. Varus and valgus stress examination under anesthesia was performed to rule out concomitant ligamentous injury.

In lateral approach group, a skin incision was centered over the lateral epicondyle, extending from the anterior aspect of the lateral column of the distal end of the humerus to approximately 2 cm distal to the radial head. Following dissection through the subcutaneous tissue layers, the lateral column is palpated. The forearm was pronated to move the radial nerve away from the surgical field. The common extensor origin in conjunction with the anterior capsule is elevated sharply as a full-thickness sleeve from the lateral supracondylar ridge anteriorly. Distally, the Kocher interval between the anconeus and extensor carpi ulnaris is identified and connected to the proximal exposure to develop a continuous full-thickness anterior soft tissue flap.

**Fig. 1:**
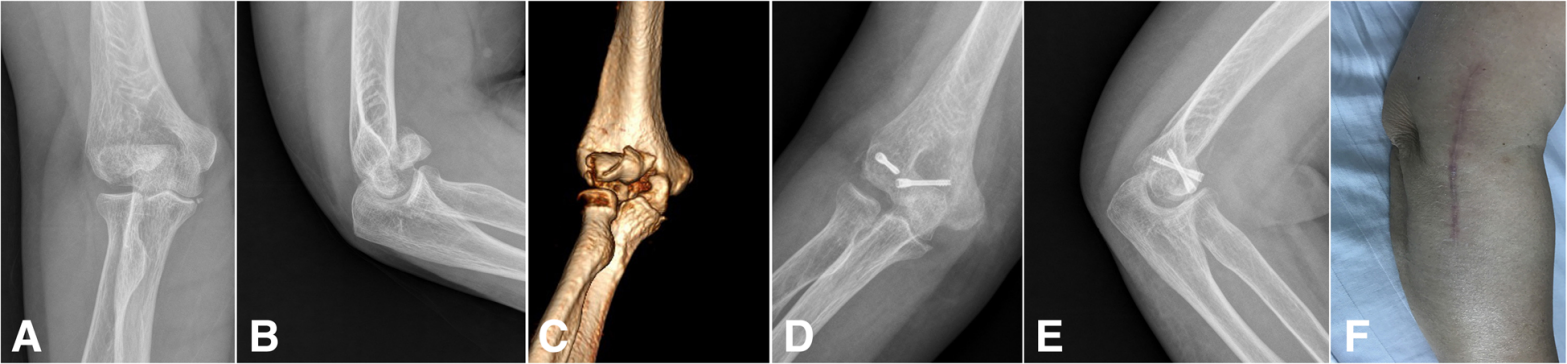
A 54-year-old female with type 3A right capitellar shear fracture from lateral approach group. **a**, **b** Anteroposterior and lateral x-ray views preoperatively. **c** 3D CT reconstruction preoperatively. **d**, **e** Anteroposterior and lateral x-ray views 21 months postoperatively showed union of the fracture fixed with two Herbert screws. **f** Incision appearance of lateral approach

The fracture site was debrided by removing blood clots, loose pieces of bone, and any interposed tissue. Saline irrigation was used to achieve greater clarity. The fracture was reduced by matching the articular fracture lines. Provisional fixation is performed with two or three guidewires for the Herbert screw. The guidewires were passed across the fracture site where the planned screw track is to be inserted. After anatomic reduction was confirmed with fluoroscopy, Herbert screws were inserted over the guidewires in anterior to posterior direction to achieve definitive fixation. The screws were buried beneath the articular surface. Upon fixation, the elbow is made to go through the full flexion-extension and rotation arc to check for the stability of fixation. Final reduction and position of the implant is checked with fluoroscopy. The common extensor origin was repaired to the soft-tissue cuff on the lateral supracondylar ridge, and the Kocher interval was closed in continuity with the proximal exposure of the lateral column. The closure of the wound is done in layers over a drain (Fig. 1).

**Fig. 2:**
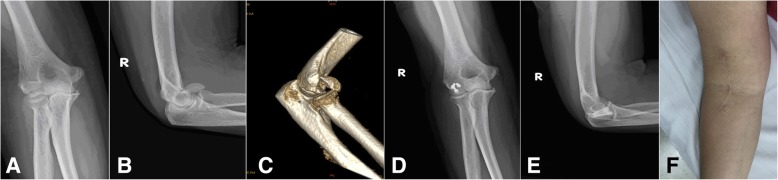
A 62-year-old female with type 1A right capitellar shear fracture from anterolateral approach group. **a**, **b** Anteroposterior and lateral x-ray views preoperatively. **c** 3D CT reconstruction preoperatively. **d**, **e** Anteroposterior and lateral x-ray views 26 months postoperatively showed union of the fracture fixed with three Herbert screws. **f** Incision appearance of anterolateral approach

In anterolateral approach group, a curved incision began 5 cm above the elbow flexion crease in the supinated forearm, and followed the lateral border of the biceps distally, but curves laterally at the elbow joint level to avoid crossing a flexion crease at 90°. Then it extended distally in the forearm along the medial border of brachioradialis. The interval was made between the brachialis and brachioradialis. The lateral cutaneous nerve of the forearm needs to be protected in the superficial plane. In the deeper plane, the radial nerve needs to be identified and protected. The brachioradialis and the radial nerve are retracted laterally and the biceps medially to expose the anterior capsule of the elbow joint. The capsule was incised to expose the capitellum. The elbow is slightly flexed to expose the capitellum and trochlea adequately. The fixation of the fracture and closure of the wound were performed in the same manner as in the lateral approach group (Fig. 2).

### Postoperative care

A long arm posterior plaster splint was applied routinely with the elbow at approximately 90° of flexion, which was kept for 2 week. Active ROM was started when the splint was removed.

Operation time, wound healing complication, and nerve injury were recorded. Clinical and radiographic evaluation was performed regularly. At each follow-up, pain, ROM, and stability of the elbow joint was assessed by clinical examination, which enabled calculation of the Mayo Elbow Performance Index (MEPI) score (90–100, excellent; 75–89, good; 60–74, satisfactory; below 60, poor). Radiographic examination was performed to evaluate the status of the bony union, heterotopic ossification, incidence of posttraumatic osteoarthritis, and avascular necrosis.

### Statistical analysis

Statistical analyses were performed with SPSS for Windows 15.0 (SPSS, USA). Count data in different groups were analyzed by chi-square test. Measurement data were expressed as mean ± standard deviation, and compared by the independent sample *T* test. Differences were considered to be significant if *p* < 0.05.

## Results

The demographics and results of the patients were presented in Table 1. Fourteen patients were assigned to lateral approach group, and 12 patients in anterolateral approach group. No significant difference was detected in the distribution of the age, gender, injury mechanism, Dubberley fracture classification, and follow-up duration between the two groups.

**Table 1:** Comparison of demographic data and clinical results between two groups

	Lateral approach group	Anterolateral approach group	*p* value
Age	43 ± 11	49 ± 10	0.512
Gender			0.259
Male	6	5	
Female	8	7	
Cause			0.193
Fall down	10	9	
Traffic accident	4	3	
Dubberley classification			0.217
1A	9	9	
2A	1	1	
3A	4	2	
Follow-up duration (month)	20 ± 5	21 ± 6	0.573
Operation time (min)	119 ± 15	93 ± 12	0.000
ROM (°) of flexion-extension	135 ± 8	133 ± 9	0.325
Loss of flexion-extension motion (°)	9 ± 2	11 ± 3	0.269
ROM of pronation-supination (°)	168 ± 9	170 ± 6	0.465
Loss of pronation-supination motion (°)	10 ± 3	8 ± 2	0.215
MEPI score	91 ± 8	92 ± 7	0.416

The operation via the anterolateral approach took significantly shorter time than via lateral approach (*p* < 0.05). There were no wound healing problems and infection for both groups. One patient from anterolateral approach group sustained incomplete posterior interosseous nerve palsy, who presented with extension deficit of his ring finger and little finger at the metacarpophalangeal joint level. It recovered completely in 4 weeks without residual compromise.

All fractures healed well in their normal anatomic position as seen on radiographs. At the final follow-up, no evidence of avascular necrosis of the fragments was found. No patients had any subjective complaints of instability of the elbow. The average ROM in the affected elbows of lateral approach group and anterolateral approach group was 135° ± 8° and 133 ± 10° in flexion-extension respectively, with an average loss of motion of 9° and 11° respectively compared with the unaffected elbows. ROM in supination-pronation in the affected elbows of lateral approach group and anterolateral approach group averaged 168° ± 7° and 170° ± 6°, with an average loss of motion of 10° and 8° respectively compared with the unaffected elbows. No significant difference was found between the two groups with respect to the ROM in supination-pronation, ROM in pronation-supination, loss of flexion-extension motion, or loss of pronation-supination motion (*p* > 0.05). The average MEPI score was more than 90 for both groups. No significant difference was found with respect to MEPI score between the two groups (*p* > 0.05). All patients were satisfied with the operative outcome and returned to their previous activity levels. No evidence of avascular necrosis, posttraumatic osteoarthritis, or heterotrophic ossification was found.

## Discussion

The choice of surgical approaches is usually based on the fracture type and complexity, comfort of the orthopedic surgeon, and protection of the blood supply. For capitellar coronal fractures, the most commonly used approach is the lateral approach of elbow joint [6–16]. This approach was characterized by anatomic safety and simplicity, and adequate exposure of the radiocapitellar compartment. However, the visualization of the trochlea and medial articular extension is limited. One surgical technique can be used to improve the exposure range. The elbow is flexed to facilitate placement of blunt Hohmann retractors deep to the brachialis and the anterior capsule and over the medial column. This can facilitate maximal exposure of the anterior aspect of distal humerus [7]. Even so, we found that the manipulation of fracture reduction and internal fixation was difficult for Dubberley type 3A fractures, because operating space for factures is confined. Inevitably, operation time would be prolonged. All the surgeries via this approach in this series obtained accurate reduction and rigid fixation. However, we think that a single lateral approach probably could not address all type 3A fractures, especially those factures with trochlea comminution. Dubberley et al. suggested that a supplemental medial-based exposure, flexor-pronator split, should be prepared to perform, if the medial aspect of the trochlea cannot be seen adequately or reduction cannot be confirmed from the lateral approach [2].

The anterolateral approach has also been used in several reports for these injuries to achieve satisfactory outcomes [17–21]. In the current study, with respect to clinical outcomes as determined by postoperative ROM of elbow and MEPI Score, no statistical difference was observed between two surgical approaches. The average MEPI score was more than 90 for both groups, which corresponds to an excellent outcome. These results are consistent with several related studies, which showed that poor results were only obtained in capitellar fractures with posterior comminution [2, 8–10]. Based on our experience, the anterolateral approach can expose the capitellum and trochlea widely by directly approaching the anterior aspect of the elbow. It makes the reduction of fracture fragments more easily and accurately. Furthermore, fixation screws can be placed more easily perpendicular to the fracture line from anterior to posterior. So the use of anterolateral approach can circumvent the disadvantages of the lateral approach, such as limited visualization of the fracture fragment and relative difficulty of putting the screws from anterior to posterior. This can explain that operation time via anterolateral approach was less in this series of patients. The disadvantage for anterolateral approach is that the dissection carries a risk of iatrogenic injury to the radial nerve. In this case series, one incomplete posterior interosseous nerve injury occur, which recovered soon without residual compromise. Furthermore, no severe never injury complications were reported in literatures for this approach. In our opinion, accurate understanding of the neurovascular anatomy of this approach is fundamental in avoiding iatrogenic injury. The incidence of radial nerve injury is very small with direct visualization and careful retraction. Hence, the anterolateral approach is a safe way to fix coronal shear fractures of capitellum.

Many types of internal fixation devices have been reported for reconstruction of capitellar fractures [22–25]. In a sawbone model biomechanical testing, Bryan and Morrey type I fractures were fixed with K-wires, Herbert screws, AO screws, and fine-threaded wires. The results showed that reconstructed fractures with screws were significantly more stable [25]. Herbert screws fixation has become popular for coronal shear fractures of the capitellum and good clinical results have been published because the advantages offered by these screws include excellent compression at the fracture fragments, stable fixation, and nonprominence of the implant intraarticularly [26].

### Limitation

The interpretation of our findings should be considered within the limitations of our study, which included a small number of cases short-term follow-up period. Furthermore, all surgeries were performed by two surgeons at a single site. The larger numbers of patients and longer follow-up period should be performed.

## Conclusion

Based on our findings, both lateral approach and anterolateral approach with Herbert screw internal fixation are suitable for coronal shear fractures of capitellum with satisfactory early outcomes. Compared with the lateral approach, the anterolateral approach made the surgical procedure easier and time saving in current series. The anterolateral approach was characterized by sufficient visualization of the joint including the medial articular surface, ease of achieving anatomic reduction and perpendicular fixation with screws in anterior to posterior direction. When the medial aspect of the trochlea is involved for capitellar coronal fractures, the anterolateral lateral approach should be preferred.

## Abbreviations

MEPI: Mayo Elbow Performance Index
ROM: Active range of motion

## References

[1] Bryan RS, Morrey BF. Fractures of the distal humerus. In: Morrey BF, edtior. The elbow and its disorders. 3rd. Philadelphia: WB Saunders; 1985. p 325–333.

[2] Dubberley JH, Faber KJ, Macdermid JC, Patterson SD, King GJ (2006). Outcome after open reduction and internal fixation of capitellar and trochlear fractures. J Bone Joint Surg Am.

[3] Trinh TQ, Harris JD, Kolovich GP, Griesser MJ, Schickendantz MS, Jones GL (2012). Operative management of capitellar fractures: a systematic review. J Shoulder Elb Surg.

[4] Goodman HJ, Choueka J (2005). Complex coronal shear fractures of the distal humerus. Bull Hosp Jt Dis.

[5] Singh AP, Singh AP (2015). Coronal shear fractures of distal humerus: diagnostic and treatment protocols. World J Orthop.

[6] Mighell M, Virani NA, Shannon R, Echols EL, Badman BL, Keating CJ (2010). Large coronal shear fractures of the capitellum and trochlea treated with headless compression screws. J Shoulder Elb Surg.

[7] Ruchelsman DE, Tejwani NC, Kwon YW, Egol KA (2008). Open reduction and internal fixation of capitellar fractures with headless screws. J Bone Joint Surg Am.

[8] Are A, Tornatore I, Theodorakis E (2016). Operative management of a shear fracture of the bilateral capitellum: a case report and review of the literature. Chin J Traumatol.

[9] Ashwood N, Verma M, Hamlet M, Garlapati A, Fogg Q (2010). Transarticular shear fractures of the distal humerus. J Shoulder Elb Surg.

[10] Singh AP, Singh AP, Vaishya R, Jain A, Gulati D (2010). Fractures of capitellum: a review of 14 cases treated by open reduction and internal fixation with Herbert screws. Int Orthop.

[11] Patterson SD, Bain GI, Mehta JA (2000). Surgical approaches to the elbow. Clin Orthop.

[12] Wang P, Kandemir U, Zhang K, Zhang B, Song Z, Huang H (2019). Treatment of capitellar and trochlear fractures with posterior comminution: minimum 2-year follow-up. J Shoulder Elb Surg.

[13] Marinelli A, Cavallo M, Guerra E, Ritali A, Bettelli G, Rotini R (2018). Does the presence of posterior comminution modify the treatment and prognosis in capitellarand trochlear fractures? Study performed on 45 consecutive patients. Injury..

[14] Sultan A, Khursheed O, Bhat MR, Kotwal HA, Manzoor QW (2017). Management of capitellar fractures with open reduction and internal fixation using Herbert screws. Ulus Travma Acil Cerrahi Derg.

[15] Ring D (2009). Open reduction and internal fixation of an apparent capitellar fracture using an extended lateral exposure. J Hand Surg Am.

[16] Claessen FM, Kachooei AR, Verheij KK, Kolovich GP, Mudgal CS (2016). Outcomes of concomitant fractures of the radial head and capitellum: the "Kissing Lesion". J Hand Microsurg.

[17] Cornelius AL, Bowen TR, Mirenda WM (2012). Anterolateral approach for an unusual pediatric capitellar fracture: a case report and review of the literature. Iowa Orthop J.

[18] Imatani J, Morito Y, Hashizume H, Inoue H (2001). Internal fixation for coronal shear fracture of the distal end of the humerus by the anterolateral approach. Shoulder Elbow Surg.

[19] Vaishya R, Vijay V, Jha GK, Agarwal AK (2016). Open reduction and internal fixation of capitellar fracture through anterolateral approach with headless double-threaded compression screws: a series of 16 patients. J Shoulder Elb Surg.

[20] Yu T, Tao H, Xu F, Hu Y, Zhang C, Zhou G (2018). Management of isolated coronal shear fractures of the humeral capitellum with Herbert screw fixation through anterolateral approach. BMC Musculoskelet Disord.

[21] Ravishankar MR, Kumar MN, Raut R (2017). Choice of surgical approach for capitellar fractures based on pathoanatomy of fractures: outcomes of surgical management. Eur J Orthop Surg Traumatol.

[22] Wolfson TS, Lowe D, Egol KA. Capitellum Fracture Open Reduction and Internal Fixation With Headless Screws.J Orthop Trauma. 2019;33Suppl 1:S5–S6. 10.1097/BOT.0000000000001529 31290817

[23] Tarallo L, Mugnai R, Adani R, Zambianchi F, Costanzini CA, Catani F (2015). Shear fractures of the distal humerus: is the use of intra-articular screws a safe treatment?. Musculoskelet Surg.

[24] Elkowitz SJ, Polatsch DB, Egol KA, Kummer FJ, Koval KJ (2002). Capitellum fractures: a biomechanical evaluation of three fixation methods. J Orthop Trauma.

[25] Koslowsky TC, Zilleken C, Dargel J, Thelen U, Burkhart KJ, Heck S (2012). Reconstruction of a Bryan and Morrey type I capitellar fracture in a sawbone model with four different fixation devices: an experimental study. Injury..

[26] Sano S, Rokkaku T, Saito S, Tokunaga S, Abe Y, Moriya H (2005). Herbert screw fixation of capitellar fractures. J Shoulder Elb Surg.

